# A Case of Cardiac Arrest during C1 Laminectomy for Irreducible Atlantoaxial Subluxation

**DOI:** 10.1155/2021/6691426

**Published:** 2021-01-18

**Authors:** Yosuke Shibao, Masao Koda, Keita Nakayama, Tomoyuki Asada, Kosuke Sato, Mamoru Kono, Fumihiko Eto, Kentaro Mataki, Hiroshi Kumagai, Katsuya Nagashima, Kousei Miura, Hiroshi Noguchi, Hiroshi Takahashi, Toru Funayama, Tetsuya Abe, Masashi Yamazaki

**Affiliations:** Department of Orthopedic Surgery, Faculty of Medicine, University of Tsukuba, 1-1-1 Tennodai, Tsukuba, Ibaraki 305-8575, Japan

## Abstract

We report a case of cardiac arrest, which occurred during C1 laminectomy for irreducible atlantoaxial subluxation, with return of spontaneous circulation (ROSC) upon interruption of the laminectomy. A 60-year-old woman with rheumatoid arthritis presented with neck pain, bilateral finger numbness, and bladder-rectal disturbance. Simple radiograph images showed that the atlantodental interval (ADI) was enlarged to 8 mm, and magnetic resonance imaging revealed severe spinal stenosis at C1. She was diagnosed with cervical spondylotic myelopathy due to atlantoaxial subluxation. Cardiac arrest occurred twice during the C1 laminectomy and occipito-cervical fusion (Occ-C3), and ROSC occurred without any treatment. There was no postoperative worsening of neurological symptoms, and the improvement of sensory and motor palsy was favorable. The pathogenic mechanism was presumed to be trigeminocardiac reflex. Cardiac arrest during upper cervical spine surgery is an important intraoperative complication of which operators should be made aware.

## 1. Introduction

Goswami et al. conducted a review of intraoperative cardiac arrest, with 7.22 cases per 10,000 occurring in noncardiac surgeries [1]. Coronary spastic angina, sick sinus syndrome, cardiovagal baroreflex, trigeminocardiac reflex, and pulmonary embolism are considered causes of intraoperative cardiac arrest [1]. Also, Timothy et al. reported that the risk of cardiac arrest for spine surgery was 21.3 per 10,000 anesthetics [2]. Cardiac arrest during cervical spine surgery is rare, and therefore, we report this case of cardiac arrest during C1 laminectomy for atlantoaxial subluxation along with a review of the literature.

## 2. Case Report

**Figure 1:**
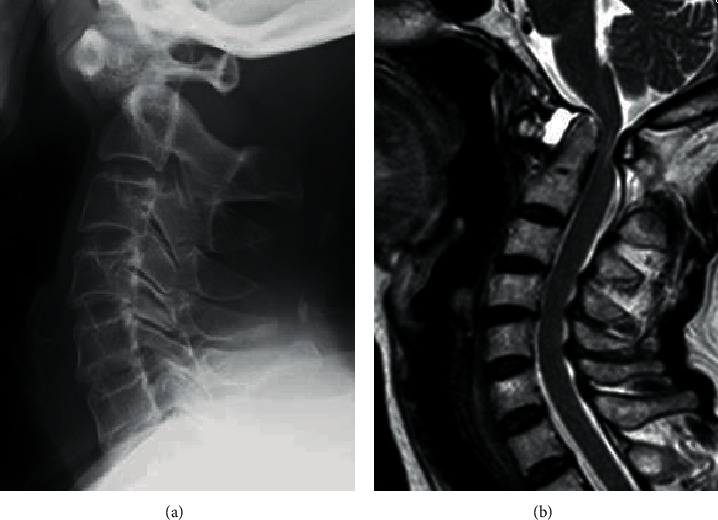
Side views of plain X-ray lateral standing images show atlantoaxial subluxation (a), and MRI confirms high compression of the spinal cord by the posterior arch of C1 (b).

**Figure 2:**
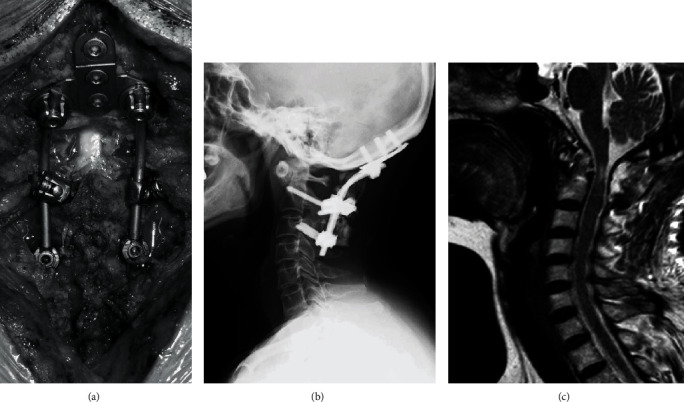
Postoperative image. Posterior decompression fusion from the occipital bone to C3 was performed (a). Side view X-rays show successful placement of the implant as indicated (b). MRI reveals that the compression of the spinal cord has been released by C1 laminectomy (c).

**Figure 3:**
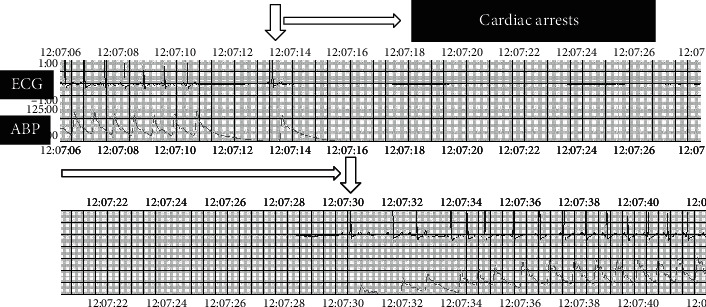
Electrocardiogram and arterial pressure findings during the first incident of cardiac arrest. ROSC resumed after approximately 15 seconds.

A 60-year-old woman with rheumatoid arthritis developed worsening neck pain and bilateral finger numbness over a 1-year duration, with urinary incontinence 3 months prior to presentation. Her functional status was limited, as she had difficulty in writing, using chopsticks, and wearing her clothes. She showed unstable gait without a cane. There were elevated deep tendon reflexes in both the upper and lower limbs. Upper and lower limb muscle strength was at a manual muscle testing level of 4. Decreased sensation was observed in both palms, and the urinary bladder-rectal function was diminished. Radiographs revealed an enlarged atlantodental interval (ADI) enlarged to 8 mm, and magnetic resonance imaging (MRI) revealed severe spinal stenosis at the C1 level (Figure 1). Upon diagnosis of irreducible C1/2 subluxation, we conducted C1 laminectomy and occipito-cervical fusion (Occ-C3) (Figure 2). Cardiac arrest occurred twice during C1 laminectomy. For the first incident, electrocardiogram (ECG) monitors suddenly flat-lined despite the lack of any obvious compression procedures on the spinal cord during the final stage of the C1 laminectomy (during excision with a 4.0 mm diamond bar) and the lack of any variations in vitals such as bradycardia. Simultaneously, we could no longer measure her arterial pressure. There was an immediate return of spontaneous circulation (ROSC) upon interruption of the surgery, without any other particular treatment (Figure 3). Motor-evoked potentials (MEP) were subsequently measured, but there were no obvious changes, and the operation was resumed upon preparation of a defibrillator. For the second incident, ECG monitors and arterial pressure suddenly flat-lined again during trimming of the cortical bone outside of the C1 with a Kerrison Rongeur, after exposing the dura at the central part of the posterior arch of C1. Cardiac arrest lasted for approximately 5 seconds, with ROSC again obtained without any specific treatment. No postoperative neurological abnormalities were noted, with walking 2 days after surgery. The patient was discharged from the hospital 2 weeks after surgery due to her ability to walk independently. Additionally, no abnormal cardiac findings were noted during evaluation by our Cardiology Department. During an outpatient visit 3 months after the operation, the patient exhibited favorable neurological recovery and continued to walk independently.

## 3. Discussion

Hoell et al. reported a case of cardiac arrest after removal of a cervical disc herniation from the back in a sitting position which was reversed by interrupting the maneuver, and the sympathetic supraspinal control system was thought to influence cardiac arrest during cervical spine surgery [3]. The system has been introduced based on clinical observations in paraplegic and quadriplegic patients [4], in which a sympathetic signal is transferred to the heart via the cervical spinal cord, although its anatomical trajectory is unknown. On the other hand, another explanation involves the trigeminocardiac reflex due to its main symptoms of sudden bradycardia, asystole, hypotension, respiratory arrest, and intestinal hypermotility occurring when the trigeminal nerve is stimulated centrally or peripherally [5]. The trigeminal spinal tract nucleus passes from the brainstem to the medulla oblongata, and in our case, it was presumed that the trigeminocardiac reflex appeared due to the additional vibrations to the spinal cord as we opened the posterior arch of C1 while it was compressing the spinal cord. When atropine is not administered, the trigeminocardiac reflex occurs in approximately 90% of strabismus and intraorbital tumor surgeries, in 1-2% of maxillary and mandibular surgeries, and in 8-18% of skull base surgeries, and both operational interruption and atropine administration are considered effective in such cases [6, 7].

## 4. Conclusion

We describe a case of cardiac arrest during C1 laminectomy for irreducible atlantoaxial subluxation. This complication can occur during upper cervical spinal surgery, and the possibility of cardiac arrest should be kept in mind during the procedure.
